# Sex Difference of Radiation Response in Occupational and Accidental Exposure

**DOI:** 10.3389/fgene.2019.00260

**Published:** 2019-05-03

**Authors:** Nadia Narendran, Lidia Luzhna, Olga Kovalchuk

**Affiliations:** ^1^Department of Biological Sciences, University of Lethbridge, Lethbridge, AB, Canada; ^2^Cumming School of Medicine, University of Calgary, Calgary, AB, Canada

**Keywords:** radiation, radiation effects and adverse reactions, sex differences, aging, cancer

## Abstract

Ionizing radiation is a well-established cause of deleterious effects on human health. Understanding the risks of radiation exposure is important for the development of protective measures and guidelines. Demographic factors such as age, sex, genetic susceptibility, comorbidities, and various other lifestyle factors influence the radiosensitivity of different subpopulations. Amongst these factors, the influence of sex differences on radiation sensitivity has been given very less attention. In fact, the International Commission on Radiological Protection (ICRP) has based its recommendations on a population average, rather than the data on the radiosensitivity of distinct subpopulations. In this study, we reviewed major human studies on the health risks of radiation exposure and showed that sex-related factors may potentially influence the long-term response to radiation exposure. Available data suggest that long-term radiosensitivity in women is higher than that in men who receive a comparable dose of radiation. The report on the biological effects of ionizing radiation (BEIR VII) published in 2006 by the National Academy of Sciences, United States emphasized that women may be at significantly greater risk of suffering and dying from radiation-induced cancer than men exposed to the same dose of radiation. We show that radiation effects are sex-specific, and long-term radiosensitivity in females is higher than that in males. We also discuss the radiation effects as a function of age. In the future, more systematic studies are needed to elucidate the sex differences in radiation responses across the life continuum – from preconception through childhood, adulthood, and old age – to ensure that boys and girls and men and women are equally protected across ages.

## Introduction

Since Henri Becquerel’s discovery of radioactivity in 1896, ionizing radiations (IRs) have been recognized to cause deleterious effects on human health (International Agency for Research on Cancer [IARC], 2000). The effects of IR exposure are dose- and dose-rate-dependent. IR exposures result in two types of health issues: deterministic and stochastic (Blakely, 2000). Deterministic effects posses a threshold dose below which the effects do not occur, and are normally observed in high doses over 0.5 Gy. These effects are seen with increasing doses of radiation to increase in severity (Blakely, 2000). Stochastic effects are known not to have a threshold dose, and are known for non-deterministic effects. These effects with increasing radiation dose increase in probability of being produced, but do not affect their severity (Blakely, 2000). Exposure of the whole body radiation (∼5 Gy and radiations stronger than X-rays or γ-rays) cause radiation sickness by inducing significant damage to the brain, gastrointestinal tract, and bone marrow, and is lethal to humans within weeks of exposure. For humans, the LD_50_ (the dose required to kill an average half of the subjects in the exposed group) is approximately 4 Gy. Exposure of the whole body to low dose rates of 0.5 Gy and above for a short period affects the gastrointestinal tract and causes nausea and vomiting. These early symptoms are followed by a latent phase (days to weeks), during which the symptoms of hair loss, gastrointestinal damage, internal bleeding, bone marrow damage, cataracts, sterility, and other radiation sickness symptoms become apparent. Doses of 20 Gy or more causes coma and rapid death due to brain damage. Partial body exposures may result in burns and damage to the eyes and skin. Tissue injury effects are thought to be caused by massive cell and tissue damage, which lead to organ failures (Mullenders et al., 2009). The vast majority of the existing data on the health risk assessment have been derived mainly from epidemiological data and case studies on populations exposed to moderate and high doses of IRs such as in the case of atomic bomb survivors in Japan and Chernobyl accident recovery operation workers (CAROWs) (Yablokov et al., 2009). The effects of lower doses of radiation are not as instantaneous, and low IR doses do not cause such apparent immediate effects. In contrast, low IR doses have been associated with late-occurring effects such as hereditary problems, cardiovascular effects, and cancer (Mullenders et al., 2009).

Currently, some studies have indicated hormesis, a biphasic dose response where an environmental agent at low dose stimulation has a beneficial effect, and at high doses leads to an inhibitory or even toxic effect (Mattson, 2008). Radiation hormesis can be considered beneficial at low doses of ionizing radiation, but harmful at high doses (Luckey, 2006). Despite this, chronic exposure to low dose ionizing radiation cannot be seen as safe.

Endorsed by authoritative scientific advisory committees such as the National Academy of Sciences’ BEIR Committees, the linear no-threshold (LNT) has been used as a radiation risk tool. Government agencies, such as U.S. Environmental Protection Agency base their radiation risk assessments and guidelines on exposure to low levels of ionizing radiation using the LNT hypothesis. This hypothesis assumes the risk of cancer due to low dose exposure is proportional to dose with no threshold dose. The threshold dose is defined as a lowest exposure level of radiation at which a specified and measurable effect manifests. However, recent research in radiobiology suggests novel damage and repair mechanisms at low doses.

A significant body of evidence on IR-induced cancers stems from the analysis of data from atomic bomb survivors and those who underwent chronic or acute occupational and accidental exposure. Several investigations have been done since 1945, which revealed elevated incidences of cancer among Hiroshima and Nagasaki A-bomb survivors. Amongst those, elevated rates of leukemia (Folley et al., 1952; Watanabe et al., 1972; Wakabayashi et al., 1983), breast cancer (Wakabayashi et al., 1983; Carmichael et al., 2003), thyroid carcinoma (Watanabe et al., 1972; Wakabayashi et al., 1983), and stomach and lung cancers (Wakabayashi et al., 1983) were reported. Increased cancer incidence has been well documented in human populations exposed to radiation from nuclear power accidents and from nuclear test sites. For example, significantly elevated mutation and cancer rates were reported in the population of the Semipalatinsk nuclear test site in Kazakhstan – the biggest nuclear testing facility in Europe (Salomaa et al., 2002; Tanaka et al., 2006). In another instance, approximately 30,000 people who lived near the Mayak nuclear facility in the southern Ural Mountains in Russia were constantly exposed to IR owing to inadequate radionuclide processing and storage. This led to an increase in the leukemia incidence rates, which were just slightly lower than that among the atomic bomb survivors in Hiroshima and Nagasaki (Kossenko, 1996a,b; Kreisheimer et al., 2003; Shilnikova et al., 2003); there were also noticeable increases in the rates of solid cancers. Considering smoking as a major confounding factor for cancer, it was found that in the Mayak facilities, among the female workers, only 3.3% were smokers, however, among the male nuclear workers 74% were smokers. These results indicate that males and females diverged in lifestyle factors that could have led to differences in radiation sensitivity. Among the males it was found that the relative risk for plutonium α-rays was 0.23/Sv (95%CI:0.16–0.31), with an inferred relative risk for smokers of 16.5 (95%CI:12.6–20.5) (Kreisheimer et al., 2003). Another nuclear catastrophe occurred in 1986 in Chernobyl, Ukraine, when the nuclear power reactor exploded releasing an enormous amount of radioactive isotopes into the environment. The largest human exposure was to iodine-131, which led to a subsequent increase in the number of thyroid carcinomas that was first noticed in 1990 (Bogdanova et al., 2006; Likhtarov et al., 2006; Williams, 2006). Several studies described significantly elevated levels of other cancers, including leukemia and lymphoma (Gluzman et al., 2005; Balonov, 2007), breast cancer (Pukkala et al., 2006; Prysyazhnyuk et al., 2007), bladder cancer (Morimura et al., 2004), and renal-cell carcinomas (Romanenko et al., 2000). Many radiation-induced non-hematological cancers continue to occur decades after exposure. Taking into consideration that only 32 years have passed since the Chernobyl catastrophe, it is too early to make final evaluations on the effects of that accident (Baverstock and Williams, 2006; Williams and Baverstock, 2006). The most recent nuclear accident happened at Fukushima, the magnitude of which was very close to that of the Chernobyl accident. Although the residing population was promptly evacuated from the areas of the strongest contamination, researchers predict that IR exposure will lead to increased cancer rates in the future.

Nowadays occupational radiation exposure is very common. The nuclear power industry, health care, and research departments, as well as defense sectors extensively utilize man-made radiations (National Commission for Security, 2012). Therefore, much attention has been given recently to the analysis of health risks in populations exposed to low or above background radiation doses (Cardis et al., 2007). Currently, the nuclear power industry employs approximately 800,000 workers worldwide. Furthermore, more than two million health care workers are exposed to radiation on a daily basis (National Commission for Security, 2012). Recent studies strongly indicate that occupational exposure to radiation lead to increased rates in IR-induced cancers. A large-scale 15-country collaborative cohort study revealed elevated cancer risks following protracted low doses of IR. Thorough analyses included 407,391 nuclear industry workers monitored individually for external radiation and 5.2 million person-years of follow-up. Importantly, a significant association was seen between IR dose and all-cause mortality, which was mainly attributable to a dose-related increase in all cancer mortality (Cardis et al., 2007; Thierry-Chef et al., 2007; Vrijheid et al., 2007).

Analyses of the outcomes in atomic bomb survivors in Hiroshima and Nagasaki and post-Chernobyl contamination areas in Belarus unambiguously demonstrate that thyroid cancer risk is increased by external radiation exposure in a dose- and age-dependent manner (Kazakov et al., 1992; Furukawa et al., 2013; Yamashita et al., 2018). However, very little attention has been given to sex differences in radiation sensitivity. In fact, the International Commission on Radiological Protection (ICRP) has based its recommendations on a population average rather than data on the radiosensitivity of a subpopulation (Hansson, 2009). Genetic factors such as gene variation have been shown to play a role in DNA damage and repair indicating a possible role in radiation sensitivity. The purpose of this review was to analyze the existing knowledge on the differences in the radiosensitivity between men and women who are at risk of radiation exposure and to identify the existing gaps in the current research literature.

## Sex Differences in Radiation Responses in Animals

Studies in animals have clearly shown that IR exposure affects males and females differently. The Kovalchuk laboratory pioneered studies on sex differences in the IR-induced gene and protein expression as well as in the global genome DNA methylation. Their findings have shown identifiable differences in the oncogenic expression in various tissues of male and female mice exposed to acute and chronic low-dose whole body irradiation and that a number of distinct pathways were affected in a sex-specific manner (Kovalchuk et al., 2004a,b; Pogribny et al., 2004; Silasi et al., 2004; Besplug et al., 2005; Cassie et al., 2006; Ilnytskyy et al., 2008; Koturbash et al., 2008a,b). Research findings have also shown alterations in IR-induced apoptosis and levels of cellular proliferation. Furthermore, with respect to the effects on the epigenome analysis revealed sex-specific disparities in DNA and histone methylation, as well as expression of DNA methyltransferases and methyl-binding proteins in IR-exposed tissues of male and female mice (Kovalchuk et al., 2004a, 2016; Pogribny et al., 2004; Koturbash et al., 2008a, 2011). Specifically, it was found that hypomethylation was increased in the liver and spleen of the female model. Sex-specific radiation responses were also seen in the brain and behavior, and these were more pronounced in females than in males (Koturbash et al., 2011; Kovalchuk et al., 2016). This allowed for the identification of microRNAs that could serve as biomarkers of brain radiation exposure in varying brain regions for the female and male models.

## Sex Differences in Radiation Effects in Humans: Unanswered Questions From Chernobyl

The NIRS (nuclear information and resources services) data indicate that radiation is more harmful for women since both cancer and death incidents were 50% higher among women than among men who had received the same radiation dose (Olson, 2011). Reproductive tissues are known to be more sensitive to IR damage, and because women have more reproductive tissues than men do, they are susceptible to more harm due to IR. Despite this difference, the same protection standards are currently applied to both men and women as per ICRP recommendations. The precise mechanisms underlying the sex differences in radiation-induced cancer remain unclear, and may include hormonal regulation, as well as genetic risks and X-linked factors that are yet to be determined (Schmitz-Feuerhake et al., 2016).

### Sex Differences in Radiation Effects: Lessons From Chernobyl

The Chernobyl nuclear reactor accident (April 26, 1986) resulted in the spread of dangerous radioactive substances across large territories of Ukraine, Belarus, Russia, and many other near-by countries. Large-scale contamination affected different groups of people such as CAROWs, people evacuated from the town of Pripyat and the 30-km zone, inhabitants from the areas of high impact in the former USSR, and contaminated European countries, and children (Prysyazhnyuk et al., 2007). Subsequently, significant effects of the radiations on health became evident in people living in the contaminated territories of Ukraine, Russia, and Belarus, and other contaminated areas in Europe. Although both men and women developed various radiation-related health complications, the effects seemed to be more evident in women, thereby affecting their reproductive abilities (Kulakov et al., 1993) and leading to increased levels of spontaneous abortions with excess female fetus losses. Moreover, the male:female fetus ratio significantly increased in areas exposed to IRs (Irgens et al., 1991).

An in-depth analysis of the populations exposed to the Chernobyl fallout also revealed changes in the sex ratio, early mortality, Down’s syndrome, and other genetic alterations. Similar results were seen in populations affected by nuclear testing or those living close to nuclear plants – all reporting marked reduction in the female birth rate (Scherb and Voigt, 2007, 2011; Schmitz-Feuerhake et al., 2016). Furthermore, a large-scale study of Israeli immigrants from Chernobyl-affected areas revealed the prevalence of bronchial asthma in adult women compared to adult men (Kordysh et al., 1995).

### Sex Differences in Radiation Effects: Thyroid Conditions

During 1993–2003, there was an increase in singular and multiple ductal goiter and thyroiditis in both men and women from contaminated regions of Belarus as compared to the non-contaminated regions. However, such increases were seen in only a select population of men (age: 50 years), while women of all ages were affected (Yablokov et al., 2009). In Poland, 50% of women living in territories contaminated by radiation from the Chernobyl accident showed increased size of the thyroid gland as compared to the women from non-contaminated areas (Yablokov et al., 2009). Hormonal disruptions were so profound that these led to disruption of the thyroid gland functions that were associated with galactorrhea (lactating) in 70-year-old women (Yablokov et al., 2009). In Ukraine, women from contaminated areas developed thyroid cancer 2.5 times more often (5 to 16 per 100,000 depending on radiation dose) than men (2 to 4 per 100,000) as indicated by cases of thyroid cancers registered from 1998 to1999. A similar trend was also observed after 1990 in Russia and Czech Republic where women were at a higher risk of developing thyroid cancer than men (Yablokov et al., 2009).

### Sex Differences in Radiation Effects: Blood-Based Diseases

In addition to thyroid gland disorders, inhabitants of nuclear-contaminated territories developed many other diseases. In 1993–2003 in Belarus, there was a significant increase in blood-based diseases among men and women, including high blood pressure and acute myocardial infarction (in women aged 35–39 years and 55–59 years), cerebrovascular diseases, and atherosclerosis (Yablokov et al., 2009). In addition, young girls (aged 10–15 years) living in regions polluted by ^137^Cs had substantial impairments in leg blood flow (Yablokov et al., 2009). In the contaminated areas of Ukraine, hormonal imbalance in children was detected during the first 2 years after the catastrophe. Both boys and girls showed increased insulin levels, and girls showed increased testosterone levels. In Belarus, an increase in testosterone levels was also observed in girls born between 1986 and 1990, which correlated with the decrease in height, weight, chest and hip size, and an increase in shoulder width and hair growth on legs. Moreover, an increase in the production of thyroglobulin in girls and a decrease in thyroxine in boys was observed at 10 years after being exposed *in utero*. Both girls and boys born in contaminated territories of Belarus suffered an increased incidence of reproductive system disruption (by five- and three-fold, respectively) (Yablokov et al., 2009).

### Sex Differences in Radiation Effects: Cancer

Cancer is a major long-term contributor to human health risks, and epidemiological studies have provided strong evidences of an increased risk of cancer development in people exposed to high levels of radiation after the Chernobyl accident. Twelve years after the disaster, the number of cancer-associated deaths in Ukraine has increased up to 18–22%; from 240 to 250 cases per 100,000 in 1985 to 255–260 cases in 1999 for men, and from 120 to 122 cases per 100,000 in 1985 to 125–130 cases in 1999 for women (Yablokov et al., 2009). In Belarus, there was a 1.5- and two-fold increase in lymphatic and hematopoietic cancers in women and men, respectively, during the first 5 years after the disaster (Yablokov et al., 2009); moreover, the overall cancer incidence rates in Belarus were higher among women (18% per year) than among men (4.4% per year). The incidence of breast cancer was significantly increased in women living in territories contaminated with ^137^Cs (185–555 kBq/m^2^) during 1990–2003 (Yablokov et al., 2009). Overall, the breast cancer incidence rates increased from 1745 to 2322 cases in Belarus during the period 1986–1999 (Yablokov et al., 2009). Similarly, there was an increase in breast cancer incidence rates among the evacuated women in Ukraine during the period 1990–2004 (Prisyazhniuk et al., 1995; Prysyazhnyuk et al., 2002b, 2007). Among female CAROWs, 41.2% of the women developed uterine fibroids, and 19% acquired mammary gland fibroadenomas at 8–9 years after the disaster (Yablokov et al., 2009). There was also a correlation between the incidence of uterine cancer and the level of radiation exposure in women living in the contaminated areas of Russia. These women showed mutations at the T-cell receptor locus in their lymphocytes; they also had an increased rate of chromosome aberrations (Yablokov et al., 2009). Cancer incidence rates among men also increased. In Ukraine, 96% of men with prostate adenoma developed precancerous lesions in the bladder urothelium (Prysyazhnyuk et al., 2002a), and there was a 1.5–2.2-fold increase in deaths caused by prostate cancer (Yablokov et al., 2009). In Russia, studies have shown differences in the predisposition to different types of cancer between male and female children, indicating that childhood cancer deaths were mainly due to leukemia in boys and brain tumors in girls (Yablokov et al., 2009).

### Sex Differences in Radiation Effects: Reproductive System-Related Conditions

In 1991, there was a 5.5-fold increase in infertility in people from contaminated areas compared to people living in non-contaminated territories, as well as a 6.6-fold increase in sperm pathologies, a two-fold increase in schlerochistosis of ovaries, and a three-fold increase in endocrine diseases. Exposure to radiations caused fetal loss. There was also a correlation between early male impotence/erectile dysfunction (in men aged 25–30 years) and levels of radiation exposure (Yablokov et al., 2009). In the affected polluted areas of Ukraine, endocrine system-related problems were observed in children. For example, 32% of girls exposed to radiation *in utero* lost their fertility compared to the controls (10.5%, *p* < 0.05) (Prysyazhnyuk et al., 2002a). In areas contaminated with ^90^Sr and Pu, there was a 2-year delay in attaining puberty in boys and a 1-year delay in girls. This is in contrast to areas contaminated with ^137^Cs where the onset of puberty occurred at an early age (Prysyazhnyuk et al., 2002a).

In 1991–2001, in the contaminated areas of Belarus, there was an increase in the number of gynecological disorders in women, including menstrual cycle disruption and pregnancy and birth complications (Yablokov et al., 2009). A significant portion of the pregnant women (54.1%) from the contaminated areas of Ukraine developed anemia and destruction of the placenta compared to the control populations (10.3%). There was also a 2.2-fold increase in the complications in labor and delivery, uterine hemorrhages, and an increased number of miscarriages and pregnancy complications (Sergienko, 1997; Lukyanova et al., 2005; Niazi and Niazi, 2011). Inhabitants and the first responders to the Chernobyl disaster were also significantly affected. Approximately 42% of the Ukrainian male CAROWs showed decreased sperm counts, 70% showed an increase in the number of dead spermatozoa, and 53% of the cases showed decreased sperm motility (Yablokov et al., 2009). In the case of Fukushima, childhood total abdominal radiation was found to inhibit follicular growth and an overall decreased oocyte count. The extent of these effects were illustrated by 1 in 6 women exposed to this form of radiation experiencing ovarian failure. However, in the case of males, the effects of the testes were dose-dependent. Increased observable effects were noted on the spermatogonia, followed by the spermatocytes and then the spermatids (Sergienko, 1997; Niazi and Niazi, 2011).

### Sex Differences in Radiation Effects: General Conditions

Health studies of CAROWs also indicated the development of cataracts, memory and psychological problems, an increase of encephalopathy, urinary system disruptions, and a significant increase in the incidence of cancer (Prisyazhniuk et al., 1995; Prysyazhnyuk et al., 2002a,b, 2007; Yablokov et al., 2009). Unfortunately, there are no statistical comparisons of the differences in the abovementioned disorders between men and women because most of the CAROWs were males.

## Sex Differences in Occupational Radiation Exposure

Occupational exposure to IR occurs in industries using radiations such as among medical workers, coal and hard-rock miners, nuclear industry workers, aircrew, and researchers in educational establishments who receive an average annual collective effective dose of ∼1000 person-Sv, <2000 and ∼2500 person-Sv, ∼1500 person-Sv, ∼800 person-Sv, and ∼30 person-Sv, respectively (Wakeford, 2006). Although the types of IRs and doses received by workers vary widely between industries, there is substantial epidemiological evidence for increased risks of cancer. The overall health risks among radiation workers in the nuclear industry have been well documented, but statistical data concerning the different levels of health risks among men and women who are occupationally exposed to IRs are lacking. The study on cancer risks among the nuclear industry employees of the Atomic Energy Authority, Atomic Weapons Establishment and British Nuclear Fuels reported that 2.6% of men and 3.7% of women developed primary infertility (Doyle et al., 2001). The authors concluded that there was no association between the low-dose radiation exposure and primary infertility in men; however, there was evidence of increased cases of primary infertility in women, although the number of women monitored in the study was too small to draw firm conclusions (Doyle et al., 2001). Another cohort study investigated the relation between cancer incidence and exposure to IR by using records from the National Dose Registry of Canada (Sont et al., 2001). This study showed a significant risk for all cancers for both sexes combined. The incidence rates of thyroid cancer, rectal cancer, leukemia, lung cancer, and melanoma increased in both sexes. There was also an increased relative risk in the cancers of the colon, pancreas, and testis in males (Sont et al., 2001).

The largest 15-country collaborative cohort study, to date, on the cancer risks from occupational exposure to low-dose IR among radiation workers in the nuclear industry showed an association between increased radiation exposure and an increase in all cancer deaths (Cardis et al., 2007), especially lung cancer and multiple myeloma. However, since the study focused mostly on men (90%), there was no evidence provided for the differences in the cancer risks between men and women. We hypothesize that the lack of data on gender differences in the sensitivity to occupational radiation exposure leads to the underestimation of health risks in certain subpopulations of radiation workers who may require stronger safety practices and regulations from nuclear industry authorities.

Perhaps the only study that evaluated adequate data on the cancer risks from IR exposure for males and females was that on workers exposed to plutonium at the Mayak nuclear facility in the Chelyabinsk region of the Russian Federation (Sokolnikov et al., 2008). Inhaled plutonium concentrates mainly in the liver and bones, with its high doses affecting the lungs, liver, and bones. The mean plutonium doses that both male and female workers received in these organs were 0.19, 0.27, and 0.98 Gy, respectively. The modifying effect of gender on cancer risks was evaluated using excess relative risk (ERR) models. The ERRs per Gy for males and females were 7.1 and 15 for lung cancer, 2.6 and 29 for liver cancer, and 0.76 and 3.4 for bone cancer, respectively (Sokolnikov et al., 2008). Such strong gender differences were also reported in an earlier study on lung cancer in Mayak workers wherein the ERR per Gy for females was about four times higher than that for males (Gilbert, 2001; Gilbert et al., 2004).

## Sex Differences in the Radiation Effects in Atomic Bomb Survivors

### Sex Differences in Radiation Effects: Cancer

Studies on cancer mortality are also available for Hiroshima and Nagasaki atomic bomb survivors. These studies demonstrate that the relative risk depends on the dose received, age at exposure, and the gender (Gilbert, 2001). According to Preston et al. (2007), increased risks for solid cancers vary with gender, and for people aged 70 years who were exposed to IR at the age of 30 years, solid cancer rates increase by about 35% per Gy for men and 58% per Gy for women. An earlier study on the same cohort estimated the excess lifetime risk per Sievert for solid cancers for those exposed at the age of 30 years to be 0.10 and 0.14 for men and women, respectively. Survivors exposed to radiation at the age of 50 years had about one-third that risk, while for those exposed at the age of 10 years, the risk was 1.0–1.8 times higher than for those exposed at the age of 30 years (Pierce et al., 2012). Cancer mortality and the excess risk for cancer were also analyzed for atomic bomb survivors exposed *in utero* and young children under 6 years of age – the analysis showed an unexplained significant difference in the mortality due to solid cancer between males and females. Specifically, nine of the deaths from solid cancer occurred in females exposed *in utero*, while there were no deaths among males. The ERR/Sv for all solid cancers in women varied from 1.6 to 17, and the ERR/Sv for female-specific cancers was 0.7–42. Gender differences existed even when female-specific cancers were excluded from the comparison (Delongchamp et al., 1997).

### Sex Differences Based on Distance From the Hypocenter

Data are also available for sex differences among atomic bomb survivors following acute radiation exposure. According to the Committee for the Compilation of Materials on Damage Caused by the Atomic Bombs in Hiroshima and Nagasaki (1981), 33.1–75% of the male atomic bomb survivors experienced symptoms of radiation sickness depending on their location and distance from the hypocenter; they also had a higher risk of sepsis after acute injuries. These gender-specific radiation responses are due to immunological and hormonal differences (Stricklin and Millage, 2012).

Overall, despite the benefits of the use of radiation for medical and industrial purposes, there are clear health risks to individuals exposed to IR, and cancer is the major long-term contributor to radiation-related deaths among the exposed people. Although health risks from radiation exposure are well understood, epidemiological studies suffer from a number of statistical uncertainties related to demographic and gender differences. Moreover, the system of human health protection from radiation exposure has not been properly addressed.

## Radiation and Aging: a Sex and Gender Link

Aging, a process of becoming older, constitutes the progressive deterioration of physiological functions and is an intrinsic process of loss of viability and increase in vulnerability. Aging is genetically determined and environmentally modulated, and is associated with an increased risk of several diseases such as cancer, stroke, and neurodegenerative, cardiovascular, and autoimmune diseases. Aging is coupled with the body’s altered capacity to withstand various stresses. At the cellular level, the cells of the aging organism significantly differ from those of the young organism at the levels of global gene expression and DNA methylation, intensity and effectiveness of DNA repair and genome maintenance mechanisms, as well as shortened telomeres (Gorbunova et al., 2007; Kovalchuk et al., 2014; Sidler et al., 2017). These lead to different stromal milieu produced by senescent cells and affect tissues, organs, and entire organisms at systemic levels (Sidler et al., 2017).

Radiation is a potent genotoxic stressor, and radiation sensitivity and the severity of the radiation effects depend upon the cellular and organisms’ capacity to effectively deal with radiation-induced damage. At the organismal level, sensitivity to radiation is often judged by the frequencies of radiation-induced cancers or other morbidities. Indeed, as described above, children living in contaminated areas have higher rates of cancers, and those exposed to therapeutic and diagnostic radiation have an increased risk of developing secondary malignancies as compared to adults (Kleinerman, 2006; Adams et al., 2010). Radiations can also cause stress-induced premature senescence, which may interfere with normal development and growth in children and young adults (Krasin et al., 2010). As reported by Brenner and Hall (Luckey, 2006), among all age groups, children show the highest radiation-induced tumor risks, which decrease with age. Growing children are much more radiosensitive, because they have a larger proportion of rapidly dividing cells (Luckey, 2006).

Furthermore, elderly individuals are also sensitive to radiation. In the elderly, increased radiation sensitivity may be due to the inability to effectively repair or replace the radiation-damaged cells (Krasin et al., 2010).While differences in radiation sensitivity are observed among the young, adult, and elderly individuals, there is only limited knowledge about the mechanisms underlying these differences at the molecular, cellular or systemic level, and the key processes may involve hormone-regulated ones, and include, but not limited to, altered levels of increased secretion of luteinizing hormone and follicle-stimulating hormone (Lannering et al., 1997), or decreased growth hormone secretion (Melin et al., 1998).

Further studies must be performed to understand the age-specific radiation effects and radiation-induced senescence and aging; moreover, these phenomena need to be analyzed in the sex and gender domain, especially in light of the potential hormonal effects mentioned above. In a recent study, we merely scanned through the sex and age-differences in radiation responses. We analyzed the incidence of γ-H2AX focus induction and persistence in the lungs, liver, spleen, thymus, and heart tissues as well as the four brain regions after exposure to 1 Gy of X-rays in very young, adolescent, young adult, and sexually mature adult male and female mice. We did not observe any sex differences in the γ-H2AX focal induction in the somatic tissues of male and female mice, with the exception of the lung tissue where the foci induction was 2 times higher in males than in females. Radiation exposure is a known risk factor for lung cancer, which is much more prevalent in males (Morita, 2002). Therefore, the molecular mechanism and the biological repercussions of the sex differences in IR-induced γ-H2AX foci formation in lung tissues should be elucidated in future studies.

## Conclusion and Outlook

**FIGURE 1:**
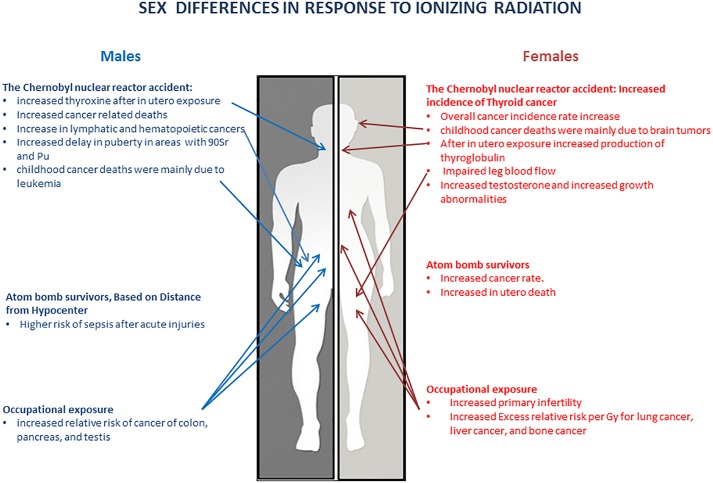
Sex differences in response to ionizing radiation.

This review summarizes the data from major human studies on the health risks of radiation exposure and shows that sex can potentially influence the prolonged response to radiation exposure (Figure 1 and Tables 1, 2). These data suggest that long-term radiosensitivity in females is higher than that in males who receive a comparable dose of radiation. Our analysis of the literature agrees with the conclusions of the recent report on the Biological effects of ionizing radiation (BEIR VII) published in 2006 by the National Academy of Sciences (NAS), United States (National Research Council, 2006). The BEIR VII report has shown that women may be at significantly greater risk of suffering and dying from IR-induced cancer than men who receive the same dose of IR. The mechanisms underlying the sex differences in IR responses are not understood. BEIR VII emphasizes several key research needs such as (i) Determination of the level of various molecular markers of DNA damage as a function of low-dose IR; (ii) Evaluation of the relevance of adaptation, low-dose hypersensitivity, and genomic instability for radiation carcinogenesis; (iii) Analysis of tumorigenic mechanisms; and (iv) Future occupational radiation studies, particularly among nuclear industry workers, including nuclear power plant workers, with special emphasis on the effects in males and females (National Research Council, 2006). Moreover, the findings of the differences in the rates of IR-induced cancers in men and women have been extensively discussed by the Multidisciplinary European Low Dose Initiative Consortium, and the need for mechanistic and systematic studies on the effects of gender on radiation risks was heavily emphasized. The re-evaluation of the existing data and the input of new data will help elucidate sex and gender differences in radiosensitivity. Overall, more studies are needed to fully elucidate the sex differences in the radiation responses across the life continuum – from preconception through childhood, adulthood, and elderhood – to ensure that boys and girls and men and women are equally protected across ages. Special attention needs to be given to the radiation effects in transgender individuals, as the health and protection of this vulnerable group is definitely lagging behind. The full in-depth analysis of the radiation effects will constitute a key step toward precision medicine and health protection.

**Table 1:** Geographic area of study, ages, and sex differences showing relationship to radiation exposure (Prysyazhnyuk et al., 2002a, 2007; Yablokov et al., 2009).

Area studied	Year	Radioactivity	Investigated ages	Diseases/Conditions	Sex differences	Statistical significance	Reference
Chernigov, Kev, Zhytomir regions	1990–1999	<100 kBq m^-2^ (low levels) 100–200 kBq m^-2^ and >200 kBq m^-2^ (medium/high levels)	Adolescents and adults	Thyroid cancer	Low levels - 2 thyroid cancer cases/year/100,000 males 5 thyroid cases/year/100,000 females (TASR) Medium/high levels – excess 4 cases/year/100,000 males 16 cases/year/100,000 females (TASR)	Regression coefficient b+ m: Males 0.04 ± 0.01 Females 0.21 ± 0.02	Prysyazhnyuk et al. (2007)
Ukraine	1986	Contaminated with Sr-90 and Pu	Adolescents	Sexual development	Puberty delayed by 2 years in boys Puberty delayed by 1 year in girls		Yablokov et al. (2009)
Belarus	1993–2003		Children born after the catastrophe in heavily contaminated areas	Reproductive organ disorders	Threefold increase in boys Fivefold increase in boys		Yablokov et al. (2009)
Ukraine	1988–1998	10–20 mSv	Adult evacuees	Thyroid gland pathology as a result of hypothyroidism, thyroidite and non-toxical nodular goiter	Annual level of thyroid pathology in females was higher than in males		Prysyazhnyuk et al. (2002a)

**Table 2:** List of most common radiation-exposure associated morbidities in females and males.

**Females**
• Thyroid cancer in women of all ages
• Goiter and thyroiditis
• Production of thyroglobulin in girls
• Testosterone increase in young females
• Impaired leg blood flow
• Reproductive system disruption
• Fertility loss
• Complications in labor and delivery, uterine hemorrhages
• Increased number of miscarriages and pregnancy complications
• Increased overall cancer incidence
• breast cancer
• Uterine fibroids and uterine cancer
• Pediatric brain tumors in girls
• Lung cancer in occupationally exposed females
**Males**
• Goiter and thyroiditis, thyroid cancer in men older than 50
• Decrease of thyroxine in boys
• Early male impotence/erectile dysfunction
• Low sperm count, decreased sperm motility
• Prostate cancer
• Pediatric leukemia in boys
• Lung cancer

## Author Contributions

OK planned the study and revised the manuscript. All authors analyzed the materials and wrote the manuscript.

## Conflict of Interest Statement

The authors declare that the research was conducted in the absence of any commercial or financial relationships that could be construed as a potential conflict of interest.
